# Identification of Pivotal MicroRNAs and Target Genes Associated with Persistent Atrial Fibrillation Based on Bioinformatics Analysis

**DOI:** 10.1155/2021/6680211

**Published:** 2021-03-06

**Authors:** Shengjue Xiao, Yufei Zhou, Qiaozhi Liu, TianTian Zhang, Defeng Pan

**Affiliations:** ^1^Department of Cardiology, The Affiliated Hospital of Xuzhou Medical University, Xuzhou, Jiangsu 221004, China; ^2^Department of Cardiology, The First Affiliated Hospital of Nanjing Medical University, Nanjing, Jiangsu 210029, China

## Abstract

Atrial fibrillation (AF) is one of the most common supraventricular arrhythmias worldwide. However, the specific molecular mechanism underlying AF remains unclear. Our study is aimed at identifying pivotal microRNAs (miRNAs) and targeting genes associated with persistent AF (pAF) using bioinformatics analysis. Three gene expression array datasets (GSE31821, GSE41177, and GSE79768) and an miRNA expression array dataset (GSE68475) associated with pAF were downloaded. Differentially expressed genes (DEGs) were identified using the LIMMA package, and differentially expressed miRNAs (DEMs) were screened from GSE68475. Target genes for DEMs were predicted using the miRTarBase database, and intersections between these target genes and DEGs were selected for further analysis, including the generation of protein–protein interaction (PPI) network, miRNA–transcription factor–target regulatory network, and drug–gene network. A total of 264 DEGs and 40 DEMs were identified between the pAF and control groups. Functional and pathway enrichment analyses of up- and downregulated DEGs were performed. The common genes (CGs) were primarily enriched in the phosphoinositide 3-kinase- (PI3K-) protein kinase B (Akt) signaling pathway, negative regulation of cell division, and response to hypoxia. The PPI network, miRNA–transcription factor–target regulatory network, and drug–gene network were constructed using Cytoscape. The present study revealed several novel miRNAs and genes involved in pAF. We speculated that miR-4298, miR-3125, miR-4306, and miR-671-5p could represent significant miRNAs that act on the target gene superoxide dismutase 2 (SOD2) during the development of pAF and may serve as essential biomarkers for pAF diagnosis and treatment. Moreover, MYC might function in pAF pathogenesis through the PI3K–Akt signaling pathway.

## 1. Introduction

Atrial fibrillation (AF) is one of the most prevalent sustained arrhythmias, estimated to affect 34 million people worldwide, and the prevalence is increasing as the population ages [1]. Based on AF duration, AF can be divided into paroxysmal AF, persistent AF (pAF), long-standing pAF, and permanent AF [2]. The morbidity rate of AF is high, leading to substantial public health and economic burdens [3]. However, the pathophysiological mechanisms of AF are complex and variable, and the pathogenesis of AF is still not fully understood [4]. Currently available drug therapies for patients with AF lack adequate efficacy and have been associated with potential adverse reactions. Although ablation is typically more effective than drug therapy, this invasive procedure has considerable potential for complications and is limited by long-term recurrence [5]. Therefore, the elucidation of the precise molecular mechanisms underlying AF is necessary for the development of novel diagnostic biomarkers and therapeutic targets.

Recently, multiple RNA families, including microRNAs (miRNAs) and long noncoding RNAs, have become the focus of investigations regarding the potential mechanism of AF [6–8]. miRNAs are small, endogenous noncoding RNAs, typically 20–25 nucleotides in length, which play core roles in the regulation of messenger RNA(mRNA) and protein expression of target genes [9], and recent studies have suggested that miRNAs may be involved in the pathophysiology of AF [10]. Xu et al. reported that miR-324-3p directly targets transforming growth factor *β*1 in fibroblasts and may be involved in myocardial fibrosis development during AF [11]. Chiang et al. identified a miR-106b-25 cluster that regulates the posttranscriptional expression of ryanodine receptor 2 (RyR2) and may serve as a potential molecular mechanism underlying the pathogenesis of paroxysmal AF [12]. Cañón et al. reported that the upregulation of miR-208b disrupts calcium dynamics in HL-1 atrial myocytes, which may contribute to atrial remodeling associated with chronic AF (CAF) [13]. Lu et al. identified the targeting of L-type Ca^2+^ channel genes by miR-328 as a contributor to adverse atrial electric remodeling in AF [14]. To date, over 200 studies have examined the involvement of various miRNAs in AF pathogenesis.

Therefore, the study of miRNAs is likely to provide useful insights into the pathophysiology of AF. Additionally, some studies have found that AF-associated miRNAs found in the circulation may serve as potential AF biomarkers, whereas tissue-specific miRNAs may represent therapeutic targets [15, 16].

In recent years, a new interdisciplinary subject known as bioinformatics has developed rapidly, combining molecular biology with information technology [17]. Using bioinformatics analysis, Zhang et al. identified potentially crucial genes associated with AF, including *CXCR4*, *IGFBP2*, *IGFBP3*, and *FHL2*, that may represent target molecules for the development of early diagnosis and future treatment strategies for AF [18]. Among these genes, CXCR4 was found to be overexpressed in CAF patients and was hypothesized to contribute to AF pathogenesis through the regulation of atrial fibrosis and structural remodeling [19]. Zhang et al. also identified miR-204-5p, miR-31-5p, and miR-223-3p as potentially significant miRNAs involved in the development of AF, which could serve as essential biomarkers for AF treatment [20]. The atrial-specific upregulation of miR-31-5p during AF in humans has been shown to be a key mechanism associated with the atrial loss of dystrophin and neuronal nitrogen oxide synthase (nNOS) [21].

To our knowledge, few studies have investigated miRNAs and their target genes in the heart tissue of patients with pAF. Therefore, we aimed to identify critical microRNAs and target genes involved in pAF using bioinformatics analysis. These results may provide novel insight into the underlying mechanisms associated with pAF pathogenesis and result in the identification of potential biomarkers for the diagnosis and treatment of pAF.

## 2. Materials and Methods

### 2.1. Microarray Data

Four pAF datasets (GSE31821, GSE41177, GSE79768, and GSE68475) were obtained from Gene Expression Omnibus (GEO, https://www.ncbi.nlm.nih.gov/geo) [22]. Three of these datasets (GSE31821, GSE41177, and GSE79768) are gene expression arrays generated using the GPL570 (HG-U133_Plus_2) Affymetrix Human Genome U133 Plus 2.0 Array (Affymetrix, Santa Clara, CA, USA). GSE68475 is an miRNA expression array generated using GPL15018 Agilent-031181 Unrestricted_Human_miRNA_V16.0_Microarray 030840. The datasets were composed as follows: GSE31821 included heart tissues obtained from 2 normal samples and 4 patients with pAF; GSE41177 contained 38 heart tissue samples, including 32 from patients with pAF and 6 normal heart tissue samples; GSE79768 consisted of 26 heart tissue samples, including 14 pAF heart tissue samples and 12 normal heart tissue samples; and GSE68475 contained 10 samples from the heart tissue of patients with pAF and 11 normal heart tissue samples.

### 2.2. Study Design and Differentially Expressed Gene Screening

**Figure 1:**
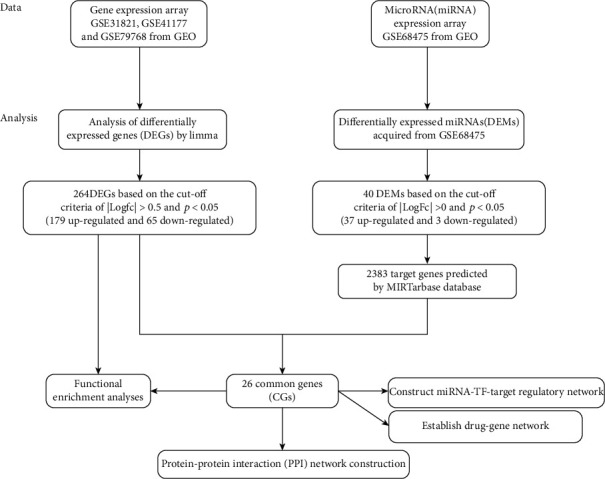
Flowchart of data analysis. GEO: Gene Expression Omnibus; mRNA: messenger RNA; TFs: transcription factors.

The research was performed according to the experimental workflow shown in Figure 1. Using the robust multiarray average algorithm in R package software, version 3.6.2 (http://www.R-project.org/), three datasets (GSE31821, GSE41177, and GSE79768) were analyzed with the Affymetrix platform. Raw array data were converted into expression values, followed by background correction, quintile normalization, and probe summarization. The GSE31821, GSE41177, and GSE79768 datasets were then merged into the integrated dataset using the ComBat algorithm of the Bioconductor sva package [23]. The LIMMA package was then applied to screen differentially expressed genes (DEGs) [24]. Differentially expressed miRNAs (DEMs) were screened from GSE68475. The cut-off used to select DEGs was defined as *p* value < 0.05, and ∣log fold − change (FC) | >0.5. DEMs were selected using the cut-off values *p* value < 0.05 and ∣log FC | >0.

### 2.3. Functional Enrichment Analysis

The online tool Database for Annotation, Visualization and Integrated Discovery (DAVID) [25] was used to annotate the Gene Ontology (GO) enrichment analysis [26] of identified DEGs. The Kyoto Encyclopedia of Genes and Genomes (KEGG) Orthology Based Annotation System (KOBAS) webserver was used to annotate and identify KEGG-enriched pathways [27]. Significant enrichment thresholds for GO and KEGG analyses were *p* value < 0.05 and count ≥ 2.

### 2.4. Protein–Protein Interaction (PPI) Network Construction

The miRTarBase database includes greater than three hundred and sixty thousand miRNA–target interactions (MTIs), which have been validated experimentally. Target genes for 40 DEMs identified from GSE68475 were predicted using the miRTarBase database [28]. A Venn diagram was used to present the intersection between identified DEGs and target genes of DEMs, referred to as common genes (CGs). Search Tool for the Retrieval of Interacting Genes (STRING, https://string-db.org) is a biological resource that provides the critical assessment and integration of protein–protein interactions [29]. In this study, the list of CGs was submitted to the STRING database to detect significant protein–protein interactions with confidence (combined score) > 0.4. Based on the STRING results, a PPI network was constructed and visualized by Cytoscape 3.7.2 software [30].

### 2.5. miRNA–Transcription Factor–Target Regulatory Network

The transcription factors (TFs) targeted by CGs were predicted using the Enrichr database (http://amp.pharm.mssm.edu/Enrichr/) [31]. The results with a *p* value < 0.05 were screened out. After miRNA–TF–target regulatory relationships were obtained, and an miRNA–TF–target regulatory network was constructed using Cytoscape software.

### 2.6. Drug–Gene Network Analysis

The Drug–Gene Interaction Database (DGIdb) [32] was developed to consolidate various data sources that reporting gene druggability and drug–gene interactions. Using the DGIdb (http://www.dgidb.org/), drug–gene pairs were predicted, and a drug–gene network was built using Cytoscape software.

### 2.7. Statistical Analysis

For all analyses, a *p* value < 0.05 was considered significant.

## 3. Results

### 3.1. Identification of DEGs

**Figure 2:**
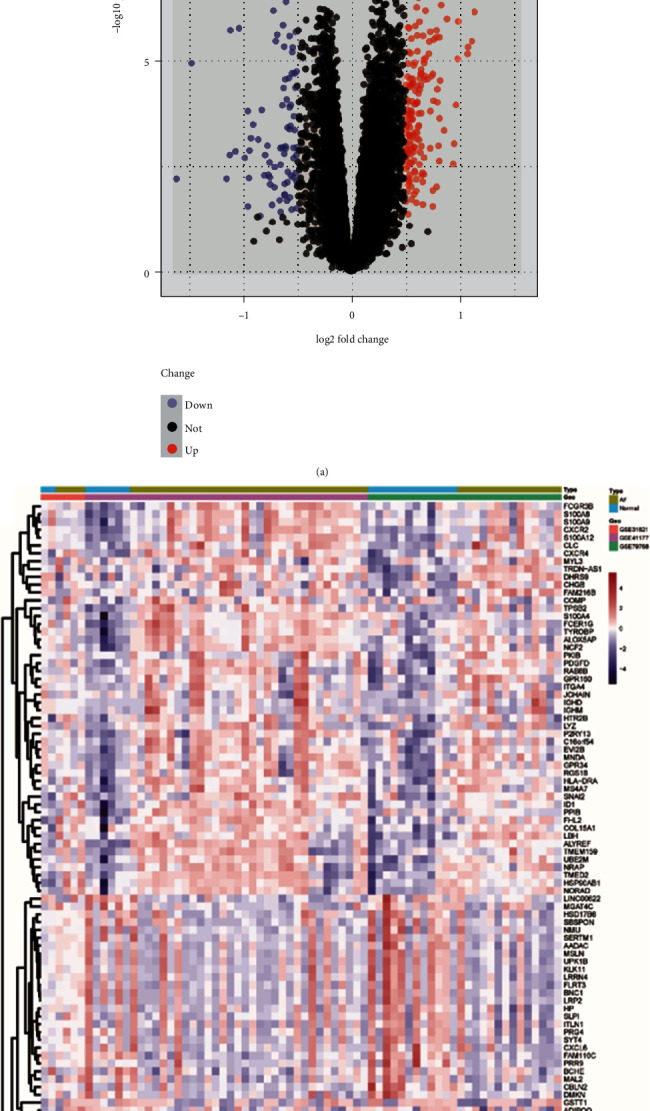
The volcano plot and heat map of DEGs. (a) Red and blue dots represent upregulated and downregulated genes, respectively. (b) The gradient color from blue to red represents the gene expression value (AF group/control group) from downregulation to upregulation, respectively. DEGs: differentially expressed genes; AF: atrial fibrillation.

A total of 264 DEGs were identified, including 179 up- and 85 downregulated genes in AF compared with normal controls (Supplementary Table 1). A total of 40 DEMs were identified between the AF group and the control group, including 37 up- and 3 downregulated miRNAs (Supplementary Table 2). A volcano plot and heat map of the identified DEGs can be observed in Figures 2(a) and 2(b), respectively.

### 3.2. Functional Enrichment Analysis

**Figure 3:**
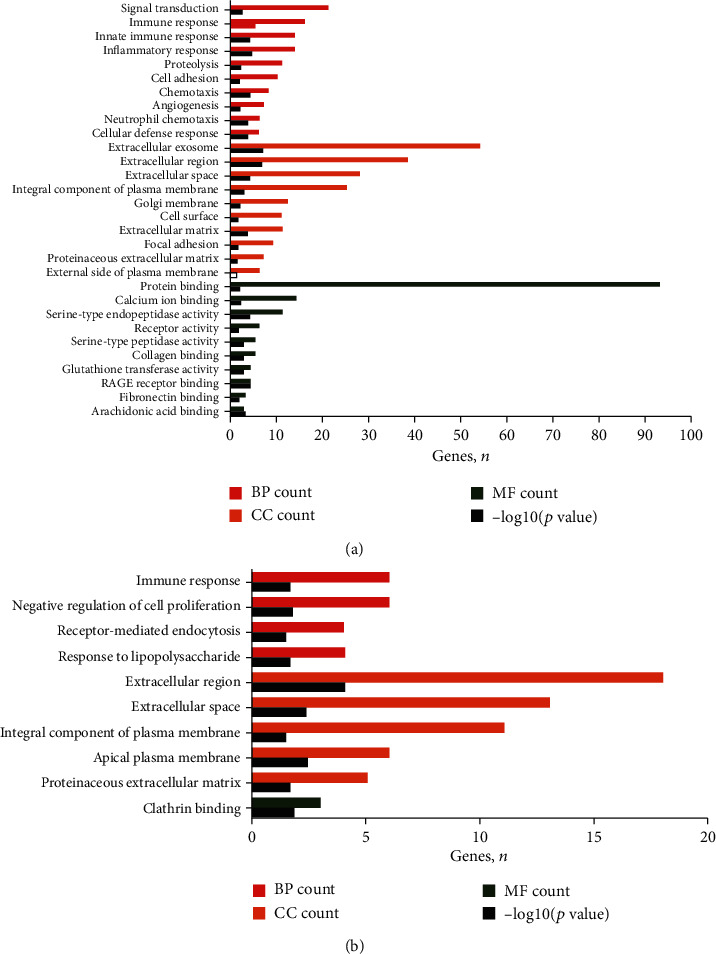
GO analysis of DEGs. (a) Upregulated DEGs. (b) Downregulated DEGs. DEGs were divided into 3 functional groups, including BP, CC, and MF. GO: Gene Ontology; BP: biological process; CC: cellular component; MF: molecular function; DEGs: differentially expressed genes.

**Figure 4:**
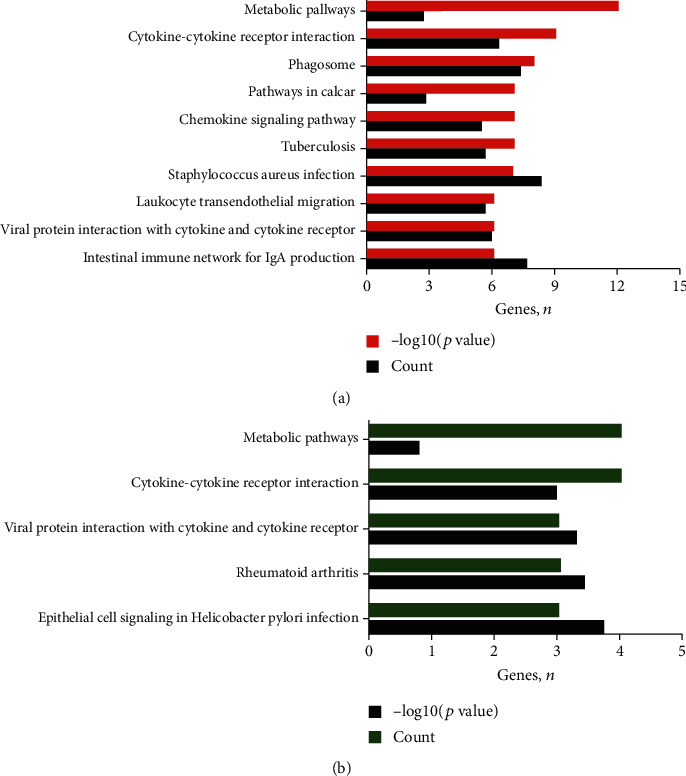
KEGG pathway analysis of DEGs. (a) Upregulated DEGs. (b) Downregulated DEGs. KEGG: Kyoto Encyclopedia of Genes and Genomes; DEGs: differentially expressed genes.

Functional enrichment analysis indicated that the upregulated DEGs were primarily involved in biological process (BP) terms, such as signal transduction and immune response. In the cell component (CC) ontology, the upregulated DEGs were significantly enriched in extracellular exosome and extracellular region. The molecular function (MF) analysis also showed that the upregulated DEGs were primarily enriched in protein binding and calcium ion binding (Figure 3(a) and Supplementary Table 3). Additionally, the KEGG pathway analysis of upregulated DEGs was found to be enriched in metabolic pathways and cytokine–cytokine receptor interactions (Figure 4(a) and Supplementary Table 4). The downregulated DEGs were primarily enriched in 10 GO terms, including 4 BP terms (negative regulation of cell proliferation), 5 CC terms (extracellular region), and 1 MF term (clathrin binding; Figure 3(b) and Supplementary Table 5). In addition, the downregulated DEGs were significantly enriched in 5 KEGG pathways, including cytokine–cytokine receptor interactions and metabolic pathways (Figure 4(b), Supplementary Table 6).

### 3.3. Target Gene Prediction

**Figure 5:**
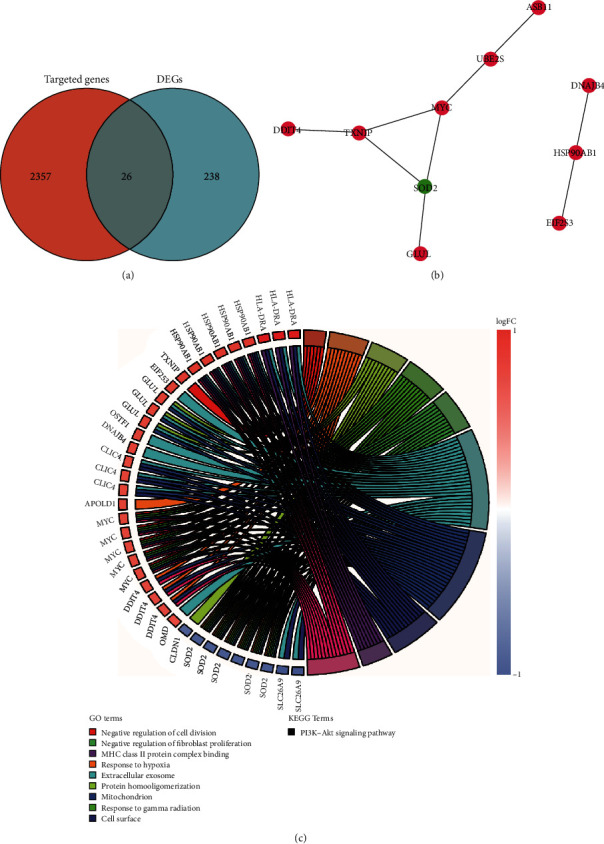
The Venn diagram, PPI network, and enrichment analyses of 26 CDEGs. (a) Venn diagram shows the intersection of DEGs and target genes of microRNAs. (b) Ellipses were used to represent nodes, and lines were used to represent edges. Red and green represent an upward adjustment and a downward adjustment, respectively. (c) Distribution of CDEGs in AF for different GO and KEGG enriched functions. GO: Gene Ontology; BP: biological process; CC: cellular component; MF: molecular function. KEGG: Kyoto Encyclopedia of Genes and Genomes; CDEGs: differentially expressed genes; AF: atrial fibrillation; PPI: protein–protein interaction.

A total of 2,383 target genes for the 40 identified DEMs were predicted by the miRTarBase database. A Venn diagram was generated to show the intersection between DEGs and these target genes (Figure 5(a)).

### 3.4. Protein–Protein Interaction (PPI) Network

To identify the most important genes, we constructed a PPI network using Cytoscape software. The PPI network included 10 nodes and 9 edges, and MYC, superoxide dismutase 2 (SOD2), and thioredoxin-interacting protein (TXNIP) had the highest numbers of nodes (Figure 5(b)). The CGs were primarily enriched in the negative regulation of cell division, the response to hypoxia, and the phosphoinositide 3-kinase– (PI3K–) protein kinase B (Akt) signaling pathway (Figure 5(c)).

### 3.5. miRNA–TF–Target Regulatory Network Analysis

**Figure 6:**
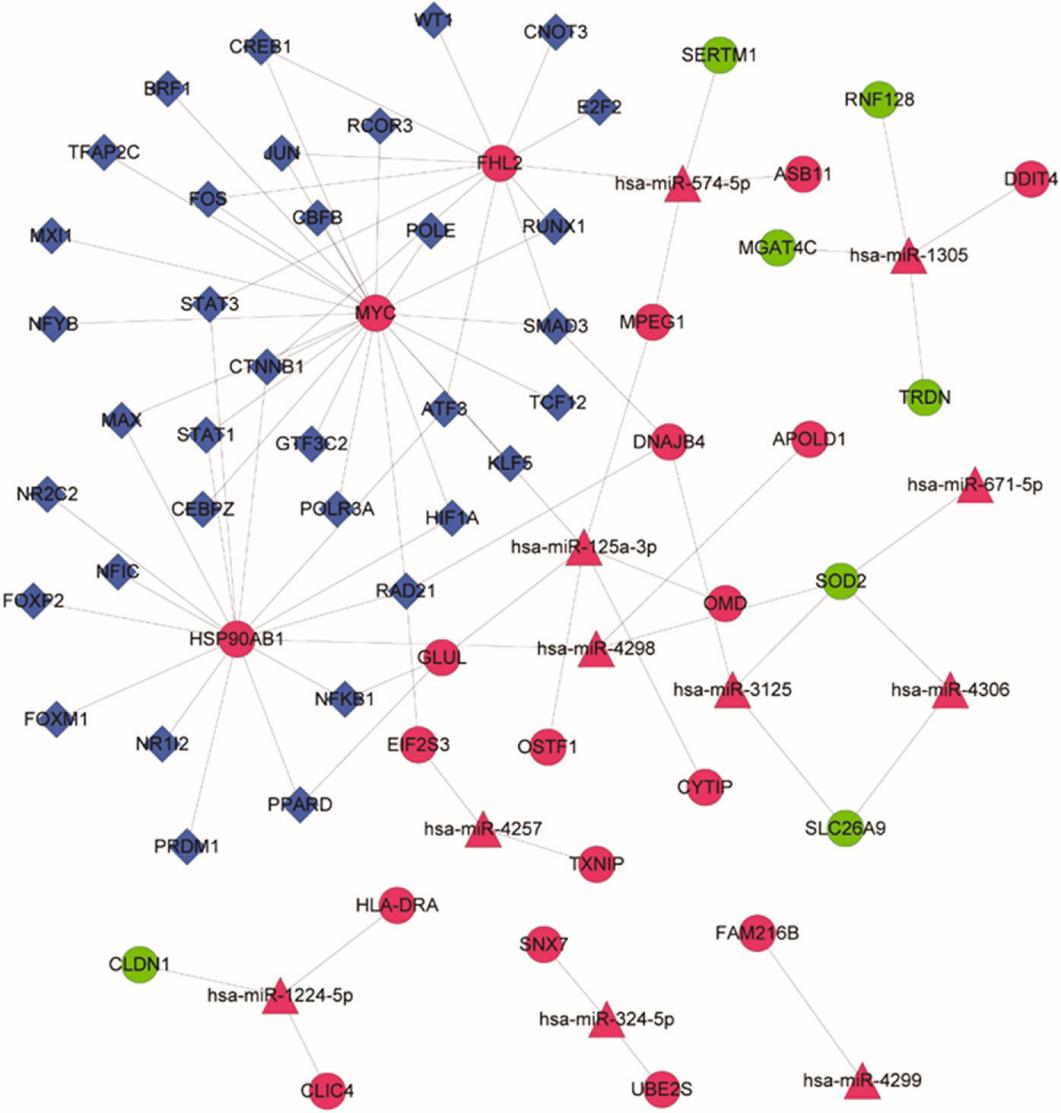
The miRNA–TF–target regulatory network. The circles represent DEGs, the triangles represent miRNAs, and the rhombi represent TFs. Red represents an upward adjustment, and green represents a downward adjustment. DEGs: differentially expressed genes; miRNA: microRNA; TFs: transcription factors.

After miRNA–gene and TF–gene pairs were predicted, 72 miRNA–TF–target regulatory relationships were obtained, and a regulatory network (involving 11 miRNAs, 35 TFs, 19 coupregulated genes, and 7 codownregulated genes) was constructed (Figure 6). In the regulatory network, SOD2 was regulated by miR-3125, miR-4306, miR-4298, and miR-671-5p. MYC and heat shock protein 90 alpha family class B member 1 (HSP90AB1) interacted with several TFs, including hypoxia-inducible factor 1*α* (HIF1*α*) and signal transducer and activator of transcription 1 (STAT1).

### 3.6. Drug–Gene Network Analysis

**Figure 7:**
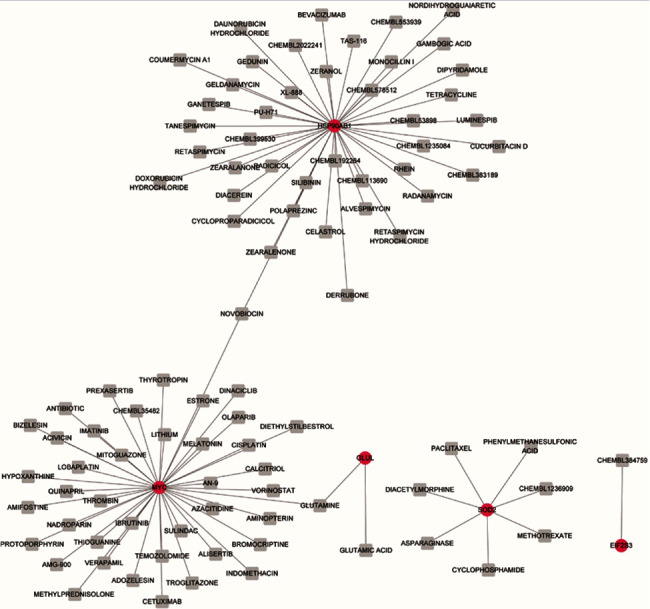
The drug–gene network. Red circle, green circle, and grey square represent upregulated DEGs, downregulated DEGs, and drugs, respectively. DEGs: differentially expressed genes.

For the CGs, 98 drug–gene pairs were acquired. The drug–gene network included 93 drugs, 4 upregulated CGs (including matrix metalloproteinase 9 (MMP9), glutamate-ammonia ligase (GLUL), eukaryotic translation initiation factor 2 subunit 3 (EIF2S3) and HSP90AB1), and 1 downregulated CG (SOD2; Figure 7). Our results revealed that dipyridamole could interact with HSP90AB1, suggesting a potential treatment target for AF, but the specific mechanism remains unclear.

## 4. Discussion

In our study, we integrated three publicly available, gene-related pAF datasets using bioinformatics analysis. We identified 264 DEGs, including 179 that were upregulated and 85 that were downregulated in pAF compared with normal controls. In addition, we identified 40 DEMs using an miRNA-related AF microarray dataset. A total of 2,383 potential target genes were associated with the identified DEMs, which were predicted using the miRTarBase database, and the intersection between these target genes and the identified DEGs was selected for further study as CGs. Finally, we constructed an miRNA–TF–target regulatory network to identify the miRNAs and TFs that regulate the expression of the identified target genes.

miRNAs can regulate the expression of target genes using multiple methods [33]. In most cases, miRNAs inhibit target mRNA expression by binding to the 3′ untranslated region (3′UTR) or protein-coding sequences to prevent translation; therefore, we were more concerned with reverse-regulated miRNA–mRNA pairs. Within the generated miRNA–TF–target regulatory network, 12 pairs of miRNA–mRNA pairs associated with pAF were identified, among which SOD2 was the most significant target gene.

Recent studies have indicated that atrial structural remodeling and electrical remodeling are important mechanisms involved in the occurrence and maintenance of AF, and atrial fibrosis, inflammation, oxidative stress, neuroendocrine, and autonomic nervous regulation have been identified as the primary factors that promote the occurrence of atrial remodeling. Macrophages also play important roles in the regulation of these factors and atrial remodeling [34, 35]. Moreover, etiological studies of familial AF have suggested that AF has a degree of heritability [36].

SOD2 is a mitochondrial antioxidant enzyme, and Xu and colleagues reported that the protein expression level of SOD2 was upregulated in AF model rats following treatment with the proliferator-activated receptor-*γ* activator pioglitazone [37]. *In vivo* (rodent) experiments have indicated the cardiac antifibrotic effects of the natural compounds bufalin and lycorine, which act by downregulating miR-671-5p [38]. Yang et al. found that miR-4306 can directly act on vascular endothelial growth factor A (VEGFA) to inhibit the extracellular signal-regulated kinase (ERK)/nuclear factor kappa B (NF-*κ*B) signaling pathway, which prevented human monocyte-derived macrophage migration [39]. Moreover, chronic, intermittent hypoxia exposure induced significant atrial remodeling in a rat model, which could be attenuated by tolvaptan, which may be due to tolvaptan-mediated alterations in the NF-*κ*B signaling pathway [40]. Brain-derived neurotrophic factor (BDNF) may affect the regeneration of human early endothelial progenitor cells by increasing the levels of miR-4298 [41]. miR-3125 can bind to identified single-nucleotide polymorphisms (SNPs) in the 3′UTR of GATA4, inducing somatic mutations and dysregulation, which may play pivotal roles in congenital heart defects (CHDs) [42]. Thus, we propose that miR-671-5p, miR-4306, miR-4298, and miR-3125 may represent significant miRNAs involved in the development of AF and play significant roles in AF, possibly through interactions with the target gene SOD2. To our knowledge, this represents the first paper to report miRNA–SOD2 pairs, which requires experimental validation.

In this study, five genes, including *FHL2*, *MYC*, *HSP90AB1*, *GLUL*, and *DNAJB4*, were targeted by TFs. Among these five genes, *MYC*, *HSP90AB1*, *GLUL*, and *DNAJB4* were also identified as hub genes in the PPI network, and MYC was significantly enriched in the PI3K–Akt signaling pathway. Studies have shown that the activation of the PI3K–Akt signaling pathway promotes the growth and proliferation of cells, inhibits apoptosis [43], reduces blood glucose levels [44], enhances inflammatory response, and aggravates the vulnerability of unstable atherosclerotic plaques [45]. Jalife and Kaur reported that the interaction between AF and atrial remodeling could exacerbate arrhythmia [46]. McMullen and collaborators found that PI3K–Akt signaling pathway inhibition increased AF incidence [47]. Xue and coworkers showed that exogenous hydrogen sulfide might reduce diabetes mellitus-induced atrial remodeling and AF through the activation of the PI3K–Akt–endothelial nitric oxide pathway [48]. Zhao and collaborators suggested that aliskiren treatment might upregulate the PI3K–Akt pathway, resulting in cardioprotective effects against rapid atrial pacing [49]. Taken together, these results suggest that regulation of the PI3K–Akt signaling pathway might participate in AF progression. Cardiac fibrosis occupies an important position in cardiac remodeling, which is consistent with AF [50]. MYC is a famous oncogene, and most studies of MYC have focused on the formation and metastasis of tumors. Zhang and Sun [51] demonstrated that the expression of c-MYC was upregulated by the long noncoding RNA ROR, which facilitated the proliferation and differentiation of cardiac fibroblasts. Moreover, MYC may represent a significant molecular factor downstream from PI3K–Akt in various tumors [52]. Based on the combination of genes that were enriched in the PI3K–Akt signaling pathway, HIF1*α* and STAT1 were identified in association with MYC. HIF1*α* expression has been shown to increase in the right atrial appendages of AF patients [53], and Tsai and coworkers demonstrated that STAT1 is activated in pigs with AF [54]. Based on our results, we speculate that HIF1*α* and STAT1 may specifically bind to MYC, regulating MYC expression, and that MYC might function in AF progression through the PI3K–Akt signaling pathway.

However, this study had some limitations. First, the miRNA and mRNA we obtained were not from identical samples. In addition, we concentrated on public databases. Additional *in vitro* and *in vivo* studies are required to validate our findings.

## 5. Conclusions

In summary, miR-4298, miR-3125, miR-4306, and miR-671-5p could represent significant miRNAs that act on the target gene SOD2 during the development of pAF and could potentially serve as essential biomarkers for pAF diagnosis and treatment. Moreover, MYC might function in the pathogenesis of AF through the PI3K–Akt signaling pathway.

## Data Availability

Research data can be obtained by contacting the first author or corresponding author: Shengjue Xiao (xiaoshengjue@126.com) or Defeng Pan (xzdefengpan@yahoo.com).

## Supplementary Materials

Supplementary Materials Supplementary Table 1: all upregulated and downregulated differentially expressed genes from the integrated dataset. Supplementary Table 2: upregulated and downregulated differentially expressed miRNAs. Supplementary Table 3: Gene Ontology analysis of the upregulated differentially expressed genes. Supplementary Table 4: Kyoto Encyclopedia of Genes and Genomes pathway analysis of upregulated differentially expressed genes. Supplementary Table 5: Gene Ontology analysis of the downregulated differentially expressed genes. Supplementary Table 6: Kyoto Encyclopedia of Genes and Genomes pathways analysis of the downregulated differentially expressed genes.
