# Supplementary material for: Identification of Pivotal MicroRNAs and Target Genes Associated with Persistent Atrial Fibrillation Based on Bioinformatics Analysis
Source: Comput Math Methods Med. 2021 Mar 6;2021:6680211. doi: 10.1155/2021/6680211 (PMC7960048; doi:10.1155/2021/6680211)
Supplement: Supplementary Materials — Supplementary Table 1: all upregulated and downregulated differentially expressed genes from the integrated dataset. Supplementary Table 2: upregulated and downregulated differentially expressed miRNAs. Supplementary Table 3: Gene Ontology analysis of the upregulated differentially expressed genes. Supplementary Table 4: Kyoto Encyclopedia of Genes and Genomes pathway analysis of upregulated differentially expressed genes. Supplementary Table 5: Gene Ontology analysis of the downregulated differentially expressed genes. Supplementary Table 6: Kyoto Encyclopedia of Genes and Genomes pathways analysis of the downregulated differentially expressed genes. [file 6680211.f1.docx]

| Supplementary Table 1. All upregulated and downregulated differentially expressed genes from the integrated dataset. | | | |
| --- | --- | --- | --- |
| gene | logFC | P.Value | adj.P.Val |
| S100A12 | 1.55708597 | 1.17E-07 | 3.07E-05 |
| CXCR2 | 1.44769418 | 9.83E-08 | 2.64E-05 |
| MNDA | 1.14258852 | 7.18E-07 | 0.000113308 |
| NRAP | 1.11684624 | 3.55E-06 | 0.00032156 |
| FHL2 | 1.07770662 | 2.89E-08 | 1.15E-05 |
| S100A8 | 1.0776275 | 4.89E-06 | 0.000390115 |
| TRDN-AS1 | 1.07313235 | 7.11E-06 | 0.000500856 |
| RGS18 | 0.98792436 | 1.21E-06 | 0.000166978 |
| CLC | 0.98196201 | 9.31E-06 | 0.00060004 |
| DHRS9 | 0.96717541 | 0.000114987 | 0.00310543 |
| LYZ | 0.94900303 | 0.000945812 | 0.012840381 |
| LBH | 0.94501761 | 2.35E-10 | 4.08E-07 |
| CHGB | 0.94076671 | 0.002873428 | 0.026289582 |
| HLA-DRA | 0.92414276 | 1.93E-07 | 4.33E-05 |
| CXCR4 | 0.91585112 | 5.67E-08 | 1.87E-05 |
| NCF2 | 0.91076784 | 8.51E-08 | 2.41E-05 |
| TMED2 | 0.883074 | 1.06E-06 | 0.000153638 |
| S100A9 | 0.8559079 | 0.000457524 | 0.00791152 |
| UBE2M | 0.85495381 | 1.29E-08 | 6.80E-06 |
| PPIB | 0.82904021 | 4.97E-07 | 8.88E-05 |
| GPR34 | 0.82019776 | 3.04E-05 | 0.001320412 |
| EVI2B | 0.81453109 | 2.87E-06 | 0.000289027 |
| FAM216B | 0.81448778 | 0.000237139 | 0.00493995 |
| MYL3 | 0.81205273 | 0.002442407 | 0.023851229 |
| ALYREF | 0.80554433 | 1.63E-13 | 2.07E-09 |
| HTR2B | 0.8045958 | 0.00106124 | 0.013911794 |
| SNAI2 | 0.80231766 | 2.50E-08 | 1.12E-05 |
| ID1 | 0.80117141 | 5.40E-08 | 1.84E-05 |
| C16orf54 | 0.79486992 | 9.90E-05 | 0.002838013 |
| TYROBP | 0.79456631 | 4.90E-05 | 0.001789931 |
| IGHD | 0.78850961 | 0.009970385 | 0.058916972 |
| MS4A7 | 0.78708322 | 1.97E-06 | 0.000224623 |
| ALOX5AP | 0.78338973 | 2.89E-06 | 0.000290548 |
| COL15A1 | 0.76173178 | 3.23E-06 | 0.000308897 |
| ITGA4 | 0.75979146 | 9.08E-05 | 0.002699175 |
| IGHM | 0.75259441 | 0.012121592 | 0.067212719 |
| TPSB2 | 0.7523394 | 2.11E-05 | 0.001017253 |
| COMP | 0.75219647 | 0.028768217 | 0.11786556 |
| TMEM159 | 0.74141879 | 2.39E-05 | 0.001104239 |
| FCER1G | 0.7300758 | 0.000160759 | 0.003854938 |
| FCGR3B | 0.72885115 | 0.00121683 | 0.015190797 |
| P2RY13 | 0.72863567 | 2.13E-06 | 0.000236805 |
| S100A4 | 0.72007318 | 3.62E-06 | 0.000322481 |
| PDGFD | 0.71789367 | 3.66E-08 | 1.36E-05 |
| GPR160 | 0.71574865 | 7.01E-06 | 0.000499281 |
| PKIB | 0.70504132 | 2.78E-05 | 0.00123918 |
| RAB8B | 0.68507523 | 6.75E-07 | 0.000111543 |
| HSP90AB1 | 0.68463292 | 9.05E-06 | 0.000592651 |
| NORAD | 0.67907071 | 2.37E-05 | 0.001102111 |
| ALDH1A1 | 0.67745529 | 2.89E-05 | 0.001260445 |
| CMTM2 | 0.67406341 | 1.16E-05 | 0.000701006 |
| CRLF1 | 0.67270564 | 0.002018354 | 0.021075706 |
| B3GALT2 | 0.67112841 | 0.005030259 | 0.038133052 |
| CMA1 | 0.67022313 | 9.77E-06 | 0.000619635 |
| MS4A4A | 0.66351736 | 2.65E-06 | 0.000276951 |
| TNFRSF17 | 0.66152808 | 0.026066332 | 0.110661232 |
| GLIPR2 | 0.6603451 | 3.46E-08 | 1.32E-05 |
| PPBP | 0.65964099 | 0.007573281 | 0.049717987 |
| BCL2A1 | 0.65867308 | 0.00459267 | 0.035911833 |
| CPA3 | 0.65243508 | 0.005190248 | 0.038924932 |
| CRISPLD1 | 0.64962953 | 0.001079922 | 0.014046648 |
| TCIM | 0.64658542 | 0.00017863 | 0.004118493 |
| COL21A1 | 0.64577299 | 1.66E-05 | 0.00087972 |
| GAPT | 0.64554368 | 2.05E-05 | 0.001000707 |
| C1QC | 0.64194314 | 2.14E-05 | 0.001021602 |
| TXNIP | 0.64131292 | 3.90E-05 | 0.001534592 |
| NPY1R | 0.63617205 | 0.001511856 | 0.017591599 |
| RPL3L | 0.63384594 | 2.67E-05 | 0.001212606 |
| INPP5A | 0.63337645 | 9.22E-10 | 1.09E-06 |
| ATP8B4 | 0.63176009 | 4.95E-05 | 0.001806381 |
| PDK4 | 0.63129286 | 0.002955052 | 0.026829529 |
| CRIP2 | 0.63009079 | 1.22E-06 | 0.000166978 |
| SELL | 0.62549271 | 0.00076254 | 0.011115236 |
| IGFBP3 | 0.62422819 | 0.000348972 | 0.006548987 |
| JAM3 | 0.6227244 | 3.32E-07 | 6.51E-05 |
| MPEG1 | 0.62218284 | 0.000184091 | 0.00419644 |
| VNN2 | 0.62076744 | 6.13E-05 | 0.002080465 |
| AMFR | 0.61913126 | 0.000377287 | 0.006894365 |
| EIF2S3 | 0.61347009 | 4.11E-05 | 0.001592427 |
| FLI1 | 0.61162586 | 4.91E-08 | 1.76E-05 |
| TPSAB1 | 0.61042699 | 6.16E-06 | 0.000458344 |
| CD37 | 0.60985895 | 9.70E-05 | 0.002810735 |
| RAB8A | 0.60942682 | 2.80E-09 | 2.09E-06 |
| IFI6 | 0.60926222 | 0.000102214 | 0.002887798 |
| PPP3R1 | 0.60873459 | 2.61E-06 | 0.000275178 |
| C1S | 0.60595724 | 0.000518113 | 0.008631225 |
| CHIC2 | 0.6036419 | 5.62E-07 | 9.95E-05 |
| PROK2 | 0.60345267 | 0.00071247 | 0.010665477 |
| CFD | 0.60173275 | 0.023713498 | 0.104180337 |
| FAP | 0.59916415 | 0.006592603 | 0.045500918 |
| CD69 | 0.59907091 | 0.012441976 | 0.06849849 |
| SLC16A9 | 0.59313284 | 0.000106612 | 0.002962509 |
| FCN1 | 0.59203912 | 5.25E-05 | 0.001871946 |
| EIF5A | 0.59129777 | 0.00051241 | 0.008564502 |
| STT3A | 0.59128696 | 2.56E-07 | 5.17E-05 |
| CLEC4A | 0.59101372 | 1.56E-06 | 0.000193526 |
| PSMC3 | 0.58844909 | 1.13E-05 | 0.000686977 |
| GLUL | 0.58780944 | 0.000109991 | 0.003014878 |
| LY96 | 0.58740462 | 0.004444473 | 0.03516921 |
| ASPN | 0.58421664 | 0.004191218 | 0.033877246 |
| OSTF1 | 0.58344995 | 6.39E-08 | 2.05E-05 |
| LYVE1 | 0.58197963 | 0.001148058 | 0.014649542 |
| DNAJB4 | 0.58137568 | 0.001146686 | 0.014649542 |
| ATP1B4 | 0.57970568 | 0.014884772 | 0.077513007 |
| GZMA | 0.57890604 | 0.002372169 | 0.023426541 |
| GDI2 | 0.57677775 | 1.32E-05 | 0.000757889 |
| C1QA | 0.57318643 | 0.001475408 | 0.017295111 |
| GSTM5 | 0.57047388 | 3.36E-05 | 0.001407536 |
| LRIF1 | 0.57015497 | 0.000577146 | 0.009272574 |
| TRIM22 | 0.56629177 | 0.000247297 | 0.005080422 |
| RNASE6 | 0.56539824 | 2.74E-05 | 0.001231951 |
| CXCL12 | 0.56363393 | 3.77E-05 | 0.001502897 |
| RAC2 | 0.56347527 | 0.000653159 | 0.01007599 |
| PDE8B | 0.56224215 | 9.46E-05 | 0.002783358 |
| SCN7A | 0.55864868 | 0.001272174 | 0.015658844 |
| CTSS | 0.5581874 | 0.000163643 | 0.003902285 |
| CLIC4 | 0.55781521 | 3.70E-05 | 0.001490553 |
| APOLD1 | 0.55773583 | 0.019772595 | 0.093483085 |
| HPGDS | 0.55753583 | 0.000257768 | 0.005236863 |
| SCOC | 0.55696771 | 0.00269999 | 0.025441198 |
| RNASE4 | 0.5560155 | 0.000186317 | 0.004228076 |
| BSG | 0.55336779 | 3.09E-06 | 0.000297861 |
| MYC | 0.55106458 | 0.009677609 | 0.057860934 |
| MAN1A1 | 0.54845285 | 0.001017357 | 0.013520529 |
| SPIN1 | 0.54841121 | 4.04E-05 | 0.001583275 |
| FBLN5 | 0.54792055 | 0.001660431 | 0.018537655 |
| TMEM256 | 0.54766138 | 2.75E-05 | 0.001233865 |
| DNAJC14 | 0.54695615 | 6.84E-06 | 0.000494949 |
| QPCT | 0.54693989 | 0.000157504 | 0.003814503 |
| CBLN1 | 0.54384785 | 0.000188985 | 0.004264636 |
| CCR2 | 0.54231269 | 0.000827411 | 0.011755057 |
| DDIT4 | 0.54188 | 0.000388185 | 0.00704883 |
| POPDC3 | 0.54185641 | 0.00276508 | 0.025775451 |
| CSF2RB | 0.54055576 | 0.000236783 | 0.00493995 |
| IGFBP2 | 0.53962813 | 0.005219927 | 0.039118425 |
| GASK1B | 0.53825213 | 0.000610333 | 0.009604419 |
| PPP1R3A | 0.53700443 | 0.007322167 | 0.048559299 |
| C1orf54 | 0.53557946 | 4.32E-05 | 0.001646026 |
| RCAN1 | 0.53533346 | 7.99E-10 | 1.01E-06 |
| MIR100HG | 0.53503973 | 0.00059293 | 0.00942341 |
| CASP1 | 0.53318946 | 1.74E-06 | 0.000206011 |
| TM6SF1 | 0.53039179 | 6.42E-06 | 0.000472397 |
| HCG11 | 0.52908114 | 0.000577295 | 0.009272574 |
| COX16 | 0.5275475 | 0.000174872 | 0.004059688 |
| ASB11 | 0.52747284 | 0.000332563 | 0.00631745 |
| HLA-DPA1 | 0.52710752 | 0.00680401 | 0.046373005 |
| DPYSL4 | 0.52706689 | 2.78E-08 | 1.15E-05 |
| BLNK | 0.5267713 | 0.008946652 | 0.055044148 |
| GATD3A | 0.52586621 | 1.79E-09 | 1.57E-06 |
| LST1 | 0.52557638 | 1.19E-05 | 0.000717567 |
| CD52 | 0.5255103 | 0.006162821 | 0.043654756 |
| TSC22D3 | 0.52459157 | 5.07E-08 | 1.76E-05 |
| CXCL11 | 0.52445383 | 0.044202563 | 0.15503173 |
| PILRA | 0.52369369 | 1.66E-06 | 0.000200138 |
| ILF2 | 0.5232555 | 1.87E-07 | 4.33E-05 |
| MRPS6 | 0.5219315 | 9.58E-05 | 0.002802245 |
| SRGN | 0.51993317 | 2.45E-05 | 0.001127991 |
| DPT | 0.51892748 | 8.75E-05 | 0.002645318 |
| EPHX1 | 0.51830623 | 0.012691875 | 0.069554633 |
| TTC37 | 0.51799989 | 0.00020981 | 0.004580852 |
| CNN1 | 0.5179608 | 0.001688944 | 0.018740447 |
| LAPTM5 | 0.51772272 | 0.001280518 | 0.015722936 |
| FMO3 | 0.51754362 | 0.003187874 | 0.028182372 |
| LCP1 | 0.51689055 | 0.000148346 | 0.003647138 |
| IFIT2 | 0.51491135 | 0.00159868 | 0.018098639 |
| UBE2S | 0.51435045 | 1.35E-08 | 6.80E-06 |
| GIMAP2 | 0.51352365 | 0.000363409 | 0.006726063 |
| SNX7 | 0.51196272 | 0.001312048 | 0.016012865 |
| CORO1A | 0.51022737 | 0.002280288 | 0.022841372 |
| CRTAM | 0.50925666 | 0.003716765 | 0.031359851 |
| CMYA5 | 0.50895069 | 0.001298678 | 0.01589664 |
| AP3S1 | 0.50854994 | 0.000302552 | 0.00589158 |
| ANP32A | 0.50851125 | 6.17E-10 | 8.90E-07 |
| GYPC | 0.50810377 | 3.86E-05 | 0.001528841 |
| OMD | 0.50428923 | 0.022007759 | 0.099839113 |
| CYTIP | 0.50426923 | 0.000899188 | 0.012407489 |
| ZBTB6 | 0.50399148 | 0.001862527 | 0.019969843 |
| FPR3 | 0.50282731 | 0.001666574 | 0.018582655 |
| TMX1 | 0.50263584 | 0.000459248 | 0.007925466 |
| IGSF5 | -0.50145411 | 0.00114231 | 0.014603908 |
| C21orf62 | -0.50196202 | 1.84E-05 | 0.000933095 |
| EPHB6 | -0.50289059 | 0.007370223 | 0.048781787 |
| MAB21L2 | -0.50289299 | 0.032328712 | 0.12691174 |
| GPC5 | -0.50587207 | 0.001764053 | 0.01927844 |
| WT1 | -0.5102231 | 0.004643257 | 0.03616721 |
| WNT2B | -0.51070438 | 2.14E-06 | 0.000236805 |
| TUFT1 | -0.51238594 | 1.06E-09 | 1.16E-06 |
| SILC1 | -0.51387951 | 4.45E-05 | 0.001672944 |
| KCNN2 | -0.51820314 | 0.000451714 | 0.00783567 |
| BLM | -0.51877692 | 2.06E-05 | 0.001000707 |
| DSC3 | -0.52657967 | 0.000211651 | 0.00460113 |
| HPR | -0.52957144 | 0.002092189 | 0.021589677 |
| IL18 | -0.53021919 | 0.005962033 | 0.042803143 |
| ANKRD36B | -0.53087114 | 0.001182292 | 0.014897917 |
| SYT13 | -0.5379038 | 1.60E-09 | 1.47E-06 |
| LINC01018 | -0.53835612 | 4.99E-06 | 0.000394594 |
| MYO5B | -0.54309077 | 6.47E-06 | 0.000474313 |
| LINC01133 | -0.54596969 | 2.06E-05 | 0.001000707 |
| GPR22 | -0.54812343 | 0.034206826 | 0.131488763 |
| MEDAG | -0.54838848 | 1.74E-07 | 4.12E-05 |
| RNF128 | -0.55232322 | 0.005412949 | 0.040103135 |
| LINC00520 | -0.55269668 | 2.05E-13 | 2.07E-09 |
| PTPRZ1 | -0.55494751 | 0.00011479 | 0.003104254 |
| EREG | -0.55875338 | 0.000358109 | 0.006652393 |
| BMP5 | -0.56391065 | 0.017630202 | 0.086608469 |
| BEX5 | -0.56601923 | 0.000426556 | 0.007535321 |
| SMTNL2 | -0.56661181 | 0.000130326 | 0.003366457 |
| HOOK1 | -0.56733567 | 2.69E-06 | 0.000277003 |
| SUSD4 | -0.57308372 | 2.66E-05 | 0.001212535 |
| MGARP | -0.58306167 | 0.004361268 | 0.034762628 |
| SLC27A6 | -0.58611367 | 0.000394418 | 0.007136311 |
| CLDN1 | -0.58635722 | 0.007698684 | 0.050181988 |
| C19orf33 | -0.59107373 | 0.021201642 | 0.097564309 |
| CD200 | -0.59322149 | 8.48E-05 | 0.00260434 |
| TMEM272 | -0.59937026 | 4.14E-07 | 7.73E-05 |
| PLLP | -0.60378245 | 0.001229505 | 0.01530169 |
| PPL | -0.60383817 | 0.001087969 | 0.014133084 |
| IER3 | -0.60599909 | 2.84E-05 | 0.001250173 |
| ALOX15 | -0.61118609 | 0.014856477 | 0.07739116 |
| LINC01844 | -0.61142479 | 0.008435325 | 0.053129642 |
| NMU | -0.61180656 | 0.015544306 | 0.079834734 |
| OTOGL | -0.61412761 | 0.001628846 | 0.018276054 |
| CBLN2 | -0.61565851 | 4.78E-06 | 0.000385917 |
| DMKN | -0.62141826 | 4.53E-05 | 0.001691447 |
| LOC100507477 | -0.62668133 | 1.48E-06 | 0.000188671 |
| MGAT4C | -0.63400688 | 0.000116648 | 0.003121028 |
| SOD2 | -0.6344489 | 0.00115036 | 0.014669265 |
| TNNI1 | -0.64957329 | 0.002974588 | 0.026946264 |
| FAM81B | -0.64971956 | 0.038854882 | 0.142987665 |
| TDRD9 | -0.65256165 | 0.001201744 | 0.015070299 |
| LINC00844 | -0.65815118 | 0.020517431 | 0.095818206 |
| WIF1 | -0.68069844 | 2.48E-06 | 0.000264975 |
| LINC00622 | -0.69400047 | 3.54E-06 | 0.00032156 |
| LRRN4 | -0.7050886 | 0.003381453 | 0.029328217 |
| CXCL6 | -0.70696411 | 0.010156241 | 0.059692939 |
| SLC26A9 | -0.71510989 | 6.74E-07 | 0.000111543 |
| HSD17B6 | -0.72753485 | 0.026567631 | 0.112011577 |
| CXCL1 | -0.74676354 | 0.008593384 | 0.053622932 |
| SERTM1 | -0.74812191 | 0.005891514 | 0.042514687 |
| TRDN | -0.75045323 | 0.002621911 | 0.024950199 |
| AADAC | -0.76155422 | 0.005149017 | 0.038745342 |
| LRRC49 | -0.77005521 | 0.001051037 | 0.013825099 |
| BCHE | -0.79231781 | 0.001755153 | 0.019194865 |
| KLK11 | -0.79966647 | 0.005285615 | 0.039493335 |
| SBSPON | -0.82317773 | 0.000149873 | 0.003678274 |
| SLPI | -0.8368494 | 0.048526854 | 0.164756101 |
| LRP2 | -0.85469873 | 0.00075458 | 0.01106315 |
| RPS11 | -0.89752565 | 8.81E-09 | 5.73E-06 |
| PRR9 | -0.9027591 | 0.000333035 | 0.006320463 |
| ETNPPL | -0.92195887 | 0.000698324 | 0.010513423 |
| FLRT3 | -0.9354454 | 0.001373258 | 0.016500364 |
| PWWP3B | -0.94026657 | 0.006134932 | 0.043544627 |
| SYT4 | -0.9505537 | 0.028918797 | 0.118279798 |
| SOSTDC1 | -0.95132762 | 0.00016028 | 0.003853977 |
| MAL2 | -0.98016786 | 0.002046781 | 0.021196997 |
| C1orf105 | -1.03108255 | 1.75E-06 | 0.000206853 |
| BNC1 | -1.06400633 | 0.001434555 | 0.016964077 |
| BEX2 | -1.1120932 | 1.96E-06 | 0.000224623 |
| UPK1B | -1.11677794 | 0.001753435 | 0.019194865 |
| MSLN | -1.14670619 | 0.006377558 | 0.044581034 |
| FAM110C | -1.1533142 | 2.56E-07 | 5.17E-05 |
| TCEAL2 | -1.18056133 | 2.63E-08 | 1.14E-05 |
| DNER | -1.47060098 | 1.17E-05 | 0.000707385 |
| PRG4 | -1.60832521 | 0.006418105 | 0.04475284 |

Abbreviations: Positive logFC values indicate upregulated expression in AF samples compared with in normal samples, whereas negative logFC values represent downregulated expression in AF samples compared with in normal samples. FC, fold change; adj.P.Value, adjusted P.Value; AF, atrial fibrillation;

| Supplementary Table 2. Upregulated and downregulated differentially expressed miRNAs. | | | |
| --- | --- | --- | --- |
| miRNA | logFC | P.value | regulation |
| Upregulated | | | |
| hsa-miR-3610 | 1.5120513 | 5.09E-04 | up |
| hsa-miR-602 | 1.2522426 | 0.014999281 | up |
| hsa-miR-3648 | 1.1632218 | 0.011070414 | up |
| hsa-miR-1181 | 1.0950499 | 0.003081342 | up |
| hsa-miR-505* | 0.879254 | 0.034647767 | up |
| hsa-miR-671-5p | 0.7892362 | 0.011599556 | up |
| hsa-miR-208b | 0.77231514 | 0.028046453 | up |
| hsa-miR-3125 | 0.70833004 | 0.02900624 | up |
| hsa-miR-4257 | 0.70426756 | 0.011457597 | up |
| hsa-miR-1973 | 0.69836694 | 0.003126202 | up |
| hsa-miR-125a-3p | 0.59157395 | 0.047900457 | up |
| hsa-miR-30d* | 0.5439254 | 0.003192564 | up |
| hsa-miR-210 | 0.53926593 | 0.001875589 | up |
| hsa-miR-499-5p | 0.52183306 | 0.006441359 | up |
| hsa-miR-3679-5p | 0.5214877 | 0.02763863 | up |
| hsa-miR-1224-5p | 0.5076376 | 0.020426916 | up |
| hsa-miR-1290 | 0.48285618 | 0.026159745 | up |
| hsa-miR-30d | 0.44573274 | 5.13E-04 | up |
| hsa-miR-4298 | 0.42112845 | 0.026426796 | up |
| hsa-miR-505 | 0.42037904 | 0.009671913 | up |
| hsa-miR-652 | 0.40417084 | 0.006855326 | up |
| hsa-miR-4291 | 0.4011467 | 0.006054637 | up |
| hsa-miR-3195 | 0.38322636 | 0.04464739 | up |
| hsa-miR-185 | 0.3773702 | 0.045186352 | up |
| hsa-miR-4306 | 0.37296 | 0.037256796 | up |
| hsa-miR-1972 | 0.37140855 | 0.007422822 | up |
| hsa-miR-324-5p | 0.3457811 | 0.043724965 | up |
| hsa-miR-574-5p | 0.31074852 | 0.027088331 | up |
| hsa-miR-24-1* | 0.30613095 | 0.04643193 | up |
| hsa-miR-30b* | 0.30416453 | 0.006061628 | up |
| hsa-miR-22 | 0.30089447 | 0.022966305 | up |
| hsa-miR-24 | 0.30055323 | 0.03340352 | up |
| hsa-miR-4299 | 0.29673588 | 0.030946381 | up |
| hsa-miR-1305 | 0.2750264 | 0.04002652 | up |
| hsa-miR-30a | 0.2542184 | 0.01706046 | up |
| hsa-miR-30e | 0.21475577 | 0.045734446 | up |
| Downregulated | | | |
| hsa-miR-100 | -1.3623356 | 0.043587588 | down |
| hsa-miR-10a | -1.5645491 | 0.030062495 | down |
| hsa-miR-31 | -2.1374257 | 0.017706852 | down |

Abbreviations: Positive logFC values indicate upregulated expression, whereas negative logFC values represent downregulated expression. FC, fold change; miRNA, microRNA.

| Supplementary Table 3. Gene Ontology analysis of the upregulated differentially expressed genes. | | | |
| --- | --- | --- | --- |
| ID | Term | Count | P-value |
| **Biological Process** | | | |
| GO:0007165 | signal transduction | 21 | 0.00440495 |
| GO:0006955 | immune response | 16 | 7.18E-06 |
| GO:0006954 | inflammatory response | 14 | 4.45E-05 |
| GO:0045087 | innate immune response | 14 | 1.58E-04 |
| GO:0006508 | proteolysis | 11 | 0.01634496 |
| GO:0007155 | cell adhesion | 10 | 0.02485393 |
| GO:0006935 | chemotaxis | 8 | 1.27E-04 |
| GO:0001525 | angiogenesis | 7 | 0.01649944 |
| GO:0006968 | cellular defense response | 6 | 2.51E-04 |
| GO:0030593 | neutrophil chemotaxis | 6 | 3.36E-04 |
| GO:0070098 | chemokine-mediated signaling pathway | 6 | 4.72E-04 |
| GO:0050900 | leukocyte migration | 6 | 0.00519078 |
| GO:0007204 | positive regulation of cytosolic calcium | 6 | 0.00766764 |
|  | ion concentration |  |  |
| GO:0042742 | defense response to bacterium | 6 | 0.01057173 |
| GO:0002250 | adaptive immune response | 6 | 0.0114785 |
| GO:0032496 | response to lipopolysaccharide | 6 | 0.01723904 |
| GO:0022617 | extracellular matrix disassembly | 5 | 0.00511476 |
| GO:0006928 | movement of cell or subcellular component | 5 | 0.0078952 |
| GO:0006956 | complement activation | 5 | 0.00821824 |
| GO:0006958 | complement activation, classical pathway | 5 | 0.01278584 |
| GO:0002523 | leukocyte migration involved in | 4 | 1.16E-04 |
|  | inflammatory response |  |  |
| GO:0008015 | blood circulation | 4 | 0.00795552 |
| GO:0019882 | antigen processing and presentation | 4 | 0.0137702 |
| GO:0009612 | response to mechanical stimulus | 4 | 0.01662065 |
| GO:0016485 | protein processing | 4 | 0.02800458 |
| GO:0050729 | positive regulation of inflammatory response | 4 | 0.02901487 |
| GO:0007160 | cell-matrix adhesion | 4 | 0.0490753 |
| GO:0002407 | dendritic cell chemotaxis | 3 | 0.01025453 |
| GO:0051493 | regulation of cytoskeleton organization | 3 | 0.01274112 |
| GO:0050832 | defense response to fungus | 3 | 0.02494217 |
| GO:0009306 | protein secretion | 3 | 0.03422205 |
| GO:0071364 | cellular response to epidermal | 3 | 0.03621625 |
|  | growth factor stimulus |  |  |
| GO:0006911 | phagocytosis, engulfment | 3 | 0.04033507 |
| **Cellular Component** | | | |
| GO:0070062 | extracellular exosome | 54 | 1.75E-07 |
| GO:0005576 | extracellular region | 38 | 2.10E-07 |
| GO:0005615 | extracellular space | 28 | 1.28E-04 |
| GO:0005887 | integral component of plasma membrane | 25 | 0.00312891 |
| GO:0000139 | Golgi membrane | 12 | 0.02295866 |
| GO:0031012 | extracellular matrix | 11 | 4.73E-04 |
| GO:0009986 | cell surface | 11 | 0.03100073 |
| GO:0005925 | focal adhesion | 9 | 0.03056939 |
| GO:0005578 | proteinaceous extracellular matrix | 7 | 0.03996309 |
| GO:0009897 | external side of plasma membrane | 6 | 0.0495526 |
| GO:0005581 | collagen trimer | 5 | 0.0108137 |
| GO:0030670 | phagocytic vesicle membrane | 4 | 0.0177025 |
| GO:0001772 | immunological synapse | 3 | 0.03993388 |
| **Molecular Function** | |  |  |
| GO:0005515 | protein binding | 93 | 0.0133709 |
| GO:0005509 | calcium ion binding | 14 | 0.01161151 |
| GO:0004252 | serine-type endopeptidase activity | 11 | 9.52E-05 |
| GO:0004872 | receptor activity | 6 | 0.04451909 |
| GO:0005518 | collagen binding | 5 | 0.00198509 |
| GO:0008236 | serine-type peptidase activity | 5 | 0.00237559 |
| GO:0050786 | RAGE receptor binding | 4 | 1.08E-04 |
| GO:0004364 | glutathione transferase activity | 4 | 0.00365441 |
| GO:0050544 | arachidonic acid binding | 3 | 7.71E-04 |
| GO:0001968 | fibronectin binding | 3 | 0.02217048 |
| GO:0015026 | coreceptor activity | 3 | 0.02899877 |

Abbreviations: DEGs, differentially expressed genes.

| Supplementary Table 4. Kyoto Encyclopedia of Genes and Genomes pathway analysis of upregulated differentially expressed genes. | | | |
| --- | --- | --- | --- |
| ID | Term | Count | P-value |
| hsa01100 | Metabolic pathways | 12 | 0.00212306 |
| hsa04060 | Cytokine-cytokine receptor interaction | 9 | 5.65E-07 |
| hsa04145 | Phagosome | 8 | 4.70E-08 |
| hsa05150 | Staphylococcus aureus infection | 7 | 4.35E-09 |
| hsa05152 | Tuberculosis | 7 | 2.23E-06 |
| hsa04062 | Chemokine signaling pathway | 7 | 3.26E-06 |
| hsa05200 | Pathways in cancer | 7 | 0.00161125 |
| hsa04672 | Intestinal immune network for IgA production | 6 | 2.21E-08 |
| hsa04061 | Viral protein interaction with cytokine | 6 | 1.14E-06 |
|  | and cytokine receptor |  |  |
| hsa04670 | Leukocyte transendothelial migration | 6 | 2.14E-06 |
| hsa04380 | Osteoclast differentiation | 6 | 4.46E-06 |
| hsa05322 | Systemic lupus erythematosus | 6 | 5.51E-06 |
| hsa04810 | Regulation of actin cytoskeleton | 6 | 7.32E-05 |
| hsa05163 | Human cytomegalovirus infection | 6 | 9.57E-05 |
| hsa04151 | PI3K-Akt signaling pathway | 6 | 0.00100875 |
| hsa05140 | Leishmaniasis | 5 | 5.32E-06 |
| hsa05133 | Pertussis | 5 | 6.02E-06 |
| hsa04650 | Natural killer cell mediated cytotoxicity | 5 | 7.36E-05 |
| hsa04514 | Cell adhesion molecules (CAMs) | 5 | 0.00012042 |
| hsa05164 | Influenza A | 5 | 0.00022085 |
| hsa05169 | Epstein-Barr virus infection | 5 | 0.00050459 |
| hsa04080 | Neuroactive ligand-receptor interaction | 5 | 0.00467818 |
| hsa04612 | Antigen processing and presentation | 4 | 0.00012818 |
| hsa04974 | Protein digestion and absorption | 4 | 0.00022763 |
| hsa04640 | Hematopoietic cell lineage | 4 | 0.00029963 |
| hsa04972 | Pancreatic secretion | 4 | 0.0003111 |
| hsa04064 | NF-kappa B signaling pathway | 4 | 0.00033499 |
| hsa04659 | Th17 cell differentiation | 4 | 0.00042896 |
| hsa05418 | Fluid shear stress and atherosclerosis | 4 | 0.00110694 |
| hsa04141 | Protein processing in endoplasmic reticulum | 4 | 0.00204114 |
| hsa04360 | Axon guidance | 4 | 0.00282878 |
| hsa05202 | Transcriptional misregulation in cancer | 4 | 0.0031122 |
| hsa04510 | Focal adhesion | 4 | 0.0039385 |
| hsa05166 | Human T-cell leukemia virus 1 infection | 4 | 0.00548088 |
| hsa04010 | MAPK signaling pathway | 4 | 0.01487193 |
| hsa05310 | Asthma | 3 | 0.00016826 |
| hsa05416 | Viral myocarditis | 3 | 0.00104466 |
| hsa04664 | Fc epsilon RI signaling pathway | 3 | 0.00147625 |
| hsa04976 | Bile secretion | 3 | 0.0017282 |
| hsa00982 | Drug metabolism - cytochrome P450 | 3 | 0.0017282 |
| hsa00980 | Metabolism of xenobiotics by cytochrome P450 | 3 | 0.00200547 |
| hsa04610 | Complement and coagulation cascades | 3 | 0.00223056 |
| hsa04662 | B cell receptor signaling pathway | 3 | 0.00247073 |
| hsa05204 | Chemical carcinogenesis | 3 | 0.00247073 |
| hsa05323 | Rheumatoid arthritis | 3 | 0.0032849 |
| hsa04658 | Th1 and Th2 cell differentiation | 3 | 0.00338429 |
| hsa04657 | IL-17 signaling pathway | 3 | 0.0034855 |
| hsa04625 | C-type lectin receptor signaling pathway | 3 | 0.00472208 |
| hsa05145 | Toxoplasmosis | 3 | 0.00590772 |
| hsa04919 | Thyroid hormone signaling pathway | 3 | 0.00678833 |
| hsa05135 | Yersinia infection | 3 | 0.00709823 |
| hsa04142 | Lysosome | 3 | 0.0074164 |
| hsa04210 | Apoptosis | 3 | 0.00968944 |
| hsa04072 | Phospholipase D signaling pathway | 3 | 0.01210989 |
| hsa04261 | Adrenergic signaling in cardiomyocytes | 3 | 0.0123258 |
| hsa04390 | Hippo signaling pathway | 3 | 0.01343849 |
| hsa04218 | Cellular senescence | 3 | 0.01484697 |
| hsa04310 | Wnt signaling pathway | 3 | 0.01484697 |
| hsa04217 | Necroptosis | 3 | 0.0153343 |
| hsa04530 | Tight junction | 3 | 0.01737334 |
| hsa04621 | NOD-like receptor signaling pathway | 3 | 0.01955648 |
| hsa05167 | Kaposi sarcoma-associated herpesvirus infection | 3 | 0.02188427 |
| hsa04020 | Calcium signaling pathway | 3 | 0.02403989 |
| hsa04015 | Rap1 signaling pathway | 3 | 0.02973593 |
| hsa05170 | Human immunodeficiency virus 1 infection | 3 | 0.03044884 |
| hsa04024 | cAMP signaling pathway | 3 | 0.03117071 |
| hsa04144 | Endocytosis | 3 | 0.04306338 |

Abbreviations: DEGs, differentially expressed genes.

| Supplementary Table 5. Gene Ontology analysis of the downregulated differentially expressed genes. | | | |
| --- | --- | --- | --- |
| ID | Term | Count | P-value |
| **Biological Process** | | | |
| GO:0008285 | negative regulation of cell proliferation | 6 | 0.01849275 |
| GO:0006955 | immune response | 6 | 0.0233805 |
| GO:0032496 | response to lipopolysaccharide | 4 | 0.0257002 |
| GO:0006898 | receptor-mediated endocytosis | 4 | 0.03540624 |
| **Cellular Component** | | | |
| GO:0005576 | extracellular region | 18 | 9.50E-05 |
| GO:0005615 | extracellular space | 13 | 0.00491287 |
| GO:0005887 | integral component of plasma membrane | 11 | 0.04312714 |
| GO:0016324 | apical plasma membrane | 6 | 0.00521092 |
| GO:0005578 | proteinaceous extracellular matrix | 5 | 0.01966177 |
| **Molecular Function** | |  |  |
| GO:0030276 | clathrin binding | 3 | 0.01612351 |

Abbreviations: DEGs, differentially expressed genes.

| Supplementary Table 6. Kyoto Encyclopedia of Genes and Genomes pathways analysis of the downregulated differentially expressed genes. | | | |
| --- | --- | --- | --- |
| ID | Term | Count | P-value |
| hsa04060 | Cytokine-cytokine receptor interaction | 4 | 0.0010558 |
| hsa01100 | Metabolic pathways | 4 | 0.16657593 |
| hsa05120 | Epithelial cell signaling in Helicobacter | 3 | 0.00018362 |
|  | pylori infection |  |  |
| hsa05323 | Rheumatoid arthritis | 3 | 0.00038693 |
| hsa04061 | Viral protein interaction with cytokine | 3 | 0.0005056 |
|  | and cytokine receptor |  |  |

Abbreviations: DEGs, differentially expressed genes.
